# Effectiveness of a Faculty Development Course on Delivering Learner-Centered Feedback Utilizing the Flipped Training Model

**DOI:** 10.51894/001c.6514

**Published:** 2018-04-27

**Authors:** Brandy Church, William D. Corser, Angela Harrison

**Affiliations:** 1 Michigan State University Statewide Campus System, College of Osteopathic Medicine, East Lansing, MI 48824

**Keywords:** resident feedback, faculty resident supervision, flipped training model

## Abstract

**CONTEXT:**

Effective feedback is an important step in the acquisition of residents’ clinical skills and a key component of most adult learning strategies. Faculty-resident feedback discussions can facilitate resident self-assessment and reflection on their performance and motivate them to study and ask questions in areas where their knowledge may be evaluated as deficient. The flipped training model approach, a type of blended learning that reverses the traditional learning environment by delivering instructional content outside of the classroom, has garnered increased support within both graduate medical education (GME) and other healthcare disciplines.

**METHODS:**

The overall purpose of this exploratory pilot project was to examine the pre-post impact of a faculty feedback flipped training model course provided to a convenience sample of community-based faculty learners. After receiving campus IRB approval, the authors developed a set of five primary course goals and objectives. A convenience sample of n = 17 community-based faculty who had completed the entire course were administered a pair of pre and post-course surveys regarding their overall feedback satisfaction and comfort levels for supervising residents.

**RESULTS:**

In summary, five of the 13 total survey items increased at statistically significant levels from pre-course levels. The majority of qualitative faculty comments also positively evaluated the flipped training model approach.

**CONCLUSIONS:**

These promising pilot findings suggest that a flipped GME faculty feedback skills training model can help improve faculty learners’ satisfaction and confidence as they supervise residents and/or medical students. The impact of these types of flipped training models for GME faculty needs to be more rigorously examined in project settings with larger samples to identify what specific types of curricular activities might prove to be most effective for diverse faculty learners in GME programs across the nation.

## INTRODUCTION

Resident physicians frequently learn through direct clinical encounters with patients under faculty supervision.^1^ As such, faculty feedback concerning resident performance is a critical element of their professional development.^1^ Effective faculty feedback helps residents improve professional behaviors and performance to meet residency program objectives.^1^ In fact, current Accreditation Council for Graduate Medical Education (ACGME) accreditation standards emphasize the necessity of direct trainee observation and provision of timely faculty feedback.^1^

Effective feedback is an important step for residents to acquire clinical skills and a key component of most adult learning strategies.^2^ Faculty-resident feedback discussions can facilitate resident self-assessment and motivate them to study in areas where their knowledge has been evaluated as deficient.^2^ Without effective feedback, residents’ medical care mistakes may go uncorrected without reinforcing their good clinical performance.^3,4^

Studies have indicated that the impact of faculty feedback messages is often related to the manner in which the feedback was provided.^5–7^ Feedback confined to an explanation of what practices should be changed can have minimal impact on subsequent behaviors.^8–10^ Specific suggestions concerning how to improve/modify practice activities or recommendations regarding the acquisition of a new skill, however, can lead to longer-term improvements in both learner knowledge and skill levels.^10,11^ On the other hand, ineffective feedback can cause learners to feel personally assaulted, or that their thoughts, opinions, and ideas are being ignored/slighted, frequently leading to defensiveness.^12,13^

The truth is that medical trainees in graduate medical education (GME) environments more often desire honest faculty feedback. For example, a study of over 1,500 residents found that 96% of respondents believed that instructor feedback was an important part of their learning, although most residents described their having received feedback as uncommon.^8^ During another study, residents indicated that feedback typically just reinforced positive behaviors or skill performance, rather than providing them constructive information for areas needing improvement.^9^ In this study, 80% of resident respondents indicated that they had either infrequently or never received corrective feedback. This finding indicates that there may be a compelling need to improve how residents are provided feedback in many GME settings.^9^

The flipped training model approach, a type of blended learning that reverses traditional didactic learning techniques with delivering online content before classroom activities, has garnered increased support in GME and other healthcare disciplines.^14–17^ Using this type of training approach, learners generally complete online content traditionally delivered in classroom lecture sessions to practice applying these concepts during classroom activities rather than completing it as assigned “homework.”

### Project Purpose

The overall purpose of this exploratory pilot project was to examine the pre-post course impact of a faculty feedback flipped training model course provided to a convenience sample of community-based faculty learners.

## METHODS

After receiving campus IRB approval, the authors, all employed by the Michigan State University Statewide Campus System (SCS)^18^ developed a set of five primary course goals and objectives for faculty participants to learn principles of providing effective learner-centered feedback to residents. These objectives included: a) describing why feedback is important for resident development; b) identifying key elements of effective feedback messages; c) describing proven strategies to provide feedback in GME settings; d) applying these principles during various teaching scenarios; and e) adapting popular feedback approaches to help create dynamic faculty-resident feedback discussions. A Feedback Course Committee consisting of three practicing clinicians and two PhD educators had consulted with the authors to make developmental curricular improvements. (Acknowledgements Section)

To meet the course objectives, the authors formulated faculty learner self-assessments and summative course evaluations aligned to each of the different objectives. Using Bloom’s taxonomy,^19^ the authors identified which objectives could be adequately covered by the online course content assignments. The authors then worked with the educator consultants to designate the objectives associated with higher-level (e.g., application, synthesis, evaluation) observable behaviors for the hands-on classroom workshop.

The online course objectives and assessments were used to create an online course using the *Desire2Learn* learning management software.^20^ This part of the course contained a series of 10-12-minute snippets of content regarding how to provide learner-centered feedback, relevant literature concerning the topic, as well as video segments for feedback visualization.

After the online course components were finalized, the authors met with the University of Michigan’s Center for Research on Learning and Teaching (CRLT) Players^21^ to plan the interactive workshop activities. This group of trained professional actors facilitated faculty development events to create instructor-learner cases during in-classroom faculty activities. During the workshop, the CRLT Players acted out resident feedback case scenarios and the authors asked faculty participants to provide feedback to the actors concerning their feedback behaviors and messages.

After the formulation of the online course and face-to-face workshop were complete, approximately 2,000 SCS-affiliated core faculty, program directors, and directors of medical education were emailed course promotion materials. Nineteen (0.95%) faculty completed the course registration process.

To evaluate the potential of the flipped training model approach to influence pre and post-course faculty learners’ feedback satisfaction and confidence levels, the authors administered a hard copy of the earlier-validated Faculty Feedback Survey (FFS)^7^ before and after the course was completed. (Appendix 1) Along with a series of items asking respondents about their GME characteristics, the FFS was comprised of a series of 13 key Likert scale items asking respondents about their current level of satisfaction with their feedback skills and overall level of confidence providing residents with feedback.

The authors sent instructions regarding how to access the online course content. Two registrants failed to complete the online course and were not invited to the subsequent in-class workshop. Each of the remaining 17 registered participants attended the event and completed the post-course survey to measure any changes in their feedback knowledge attainment.

## RESULTS

### Descriptive Statistics

A total sample of 17 faculty respondents completed over 95% of FFS^7^ survey items in both pre and post-course surveys. Nine (47%) sample respondents reported their gender as Male, nine (47%) participants were Female, and one (5%) faculty reported their gender as *Transgender* at time of the post-course survey. Six faculty indicated that they had been supervising residents and/or medical students for between *Zero and Five Years*, with six reporting *Six to Ten Years* and the remaining respondents reporting *11 years or More.* Only two (11%) faculty indicated that they had received any prior formal feedback training.

Two (11%) faculty indicated before the course that they “rarely” ever provided such feedback to residents/students, four (21%) indicated *Not Every Day* to this survey item, with the remaining 11 (58%) of faculty indicating *Not Every Day but Every 2 to 5 Days*. A total of 15 (88%) of sample faculty indicated that they had generally “enjoyed providing feedback” to residents/medical students.

Each of the 17 survey respondents indicated at least two “barriers” to their providing either *positive feedback* and/or *constructive criticism* to residents/students (from a total of 12 provided barriers). The Mean number of barriers reported by participants was 3.94 (SD 1.89) and ranged from two to seven. Cited barriers included: a) *Don’t Feel Skilled at Giving Feedback.* (n = 6), b) *Residents/medical students don’t accept feedback well.* (n = 5), c) *I don’t want to harm my rapport with residents/students.* (n = 16), d) *Time constraints.* (n = 11), e) *I don’t think of it during shift.* (n = 5) and f) *Lack of a Private Place to* *Talk.* (n = 4).

### Pre and Post-Course Survey Response Patterns

The analyst author (WDC) generated descriptive analyses of the working data set to evaluate appropriate analytic options and compare pre-to-post-course feedback response data. As might be readily expected from a smaller self-selected sample, he confirmed that the distribution of summary and individual FFS^7^ score patterns was not normally distributed. In response, a series of non-parametric Wilcoxon Matched Pair t test analytic procedures^22^ were completed using SPSS Version 22^23^ analytic software. The authors used these procedures to compare potential pre-post differences in individual-item and composite pre-post course respondent feedback satisfaction and confidence scores for statistical significance observing a two-tailed coefficient Alpha level of 0.05.

### Pre-Course Surveys

A total convenience sample of 17 faculty completed the FFS^7^ both before, and after they completed the flipped curriculum feedback course to be included in analyses. With regard to the three (i.e. *quality of feedback* (QF), *amount of feedback* (AF), *timeliness of* *feedback* (TF) “summary” feedback **satisfaction** items, the sample pre-course means were obtained: a) QF Mean 3.47 (SD 0.624), b) AF Mean 3.18 (SD 0.728), and c) TF Mean 3.47 (SD 0.717). (on possible five-point Likert scale from “1” (“*Very Dissatisfied”*) to”5” (“*Very Satisfied”*). These initial findings demonstrated that the average course participant felt a moderate level of pre-workshop satisfaction with the feedback patterns they currently provided residents and students. (See Table 1 for all summary and individual pre and post-course respondent scores).

**Table 1:** Descriptive Statistics of Faculty Feedback Survey 7 Scores (N = 17 GME Faculty)

	**Pre-Course Mean** **(SD)** **(range)**	**Post-Course Mean** **(SD)** **(range)**
**I. Summary Feedback Scores** (possible range 1 *Very Dissatisfied* through 5 *Very Satisfied*)		
Quality of (My) Feedback to Residents/Medical Students	3.47 (SD 0.624) (2 to 4)	4.00 (0.612) (3 to 5)
Amount of (My) Feedback to Residents/Medical Students	3.18 (0.728) (2 to 4)	3.76 (0.903) (2 to 5)
Timeliness of (My) Feedback to Residents/Medical Students	3.47 (0.717) (2 to 5)	3.88 (0.928) (2 to 5)
		
**II. Individual Feedback Satisfaction Scores** (possible range 0 Poor through 5 Excellent)		
1. “Quality of (My) Positive Feedback”	4.00 (0.730)	4.25 (0.577)
2. “Quality of (My) Constructive Criticism”	3.31 (0.704)	3.69 (0.793)
3. “Quality of Feedback regarding Medical Knowledge”	3.14 (0.770)	3,57 (0.646)
4. “Quality of Feedback re: Communication Skills”	3.27 (0.961)	3.87 (0.915)
5. “Quality of Feedback re: Professionalism”	3.27 (0.961)	3.73 (0.961)
6. “Quality of Feedback re: Procedural Skills”	3.27 (1.104)	3.82 (1.178)
7. “Quality of Feedback re: Documentation”	3.46 (0.967)	3.85 (0.801)
8. “Quality of Feedback re: Evidence-Based Decision making”	3.47 (0.834)	3.73 (0.961)
		
**III. Summary Comfort Scores** (poss. range from 1 Extremely Uncomfortable through 8 Extremely Comfortable)		
1. “Comfort Providing Positive Feedback”	6.29 (1.359) (4 to 8)	6.59 (0.030) (5 to 8)
2. “Comfort Providing Constructive Criticism”	5.18 (1.629) (2 to 8)	6.00 (1.414) (3 to 8)

The next eight individual pre-course feedback satisfaction item ratings data were fairly similar to the summary pre-course measures, (although each item on a five point “0” (“*Poor”*) to”5” (“*Excellent”*) scale). Participants’ self-rating scores each averaged in the”3” (*Good)* or “4” (“*Very Good”*) rating categories. Two remaining **comfort** survey items (on an eight-point Likert scale from”1” (“*Extremely Uncomfortable”*) to”8” (“*Extremely Comfortable”*) averaged slightly higher than the satisfaction items in the 5 or 6 categories. (Table 1).

### Comparison of Pre-to-Post-Course Feedback Satisfaction and Comfort Levels

Each of the three (i.e. QF, AF, TF) pre-to-post-course feedback satisfaction ratings scores were found to have increased at statistically significant levels (t scores between = 2.135 and 2.729, p values ranging from 0.015 to 0.049). (Table 1). Overall, however, the majority of individual feedback satisfaction (n = 8) or comfort (n = 2) score increases failed to reach statistical significance except for one item (i.e., *Quality of Feedback to Residents related to Procedural* *Skills*) (t = 2.631, p = 0.025). (Table 2)

**Table 2:** Pre and Post-Course Faculty Feedback Survey 7 Scores (N = 17 GME Faculty with complete data) *

	**Pre-Course Mean Score**	**Post-Course** **Mean** **Score**		**Difference**	**Z Score**	**Significance**
**I. Summary Feedback Scores** (poss. range 1 through 5)						
1. Quality of My Feedback	3.47	4.00		+ 0.529	2.729	**0.015**
2. Amount of My Feedback	3.18	3.76		+ 0.588	2.582	**0.020**
3. Timeliness of My Feedback	3.47	3.88		+ 0.412	2.135	**0.049**
						
						
**II. Individual Feedback Satisfaction Scores** (poss. range 0 through 5)						
1. Quality of (My) Positive Feedback”	4.00	4.25		+ 0.025	1.464	0.164
2. Quality of (My) Constructive Criticism”	3.31	3.69		+ 0.375	2.087	0.054
3. Feedback regarding Medical Knowledge”	3.14	3.57		+ 0.429	1.710	0.111
4. Feedback re: Communication Skills”	3.27	3.87		+ 0.600	1.964	0.070
5. Feedback re: Professionalism”	3.27	3.73		+ 0.467	1.974	0.068
6. Feedback re: Procedural Skills”	3.27	3.82		+ 0.545	2.631	**0.025**
7. Feedback re: Documentation”	3.46	3.85		+ 0.385	1.806	0.096
8. Feedback re: Evidence-Based Decision making”	3.47	3.73		+ 0.267	1.468	0.164
						
**III. Summary Comfort Scores** (poss. range 1 through 8)						
1. Comfort Providing Positive Feedback	6.29	6.59		+ 0.294	1.429	0.172
2. Comfort Providing Constructive Criticism	5.18	6.00		+ 0.824	2.190	**0.044**

*Series of Wilcoxon Matched Pair Tests

Statistically Significant Differences at Alpha of less than 0.05 are listed in Bold font

Readers should note that only two of these eight individual survey items had complete participant data, indicating that respondents may have experienced some difficulty answering some of these items. In addition, one of the two final feedback comfort survey items with complete survey data (i.e. *Overall Comfort* *with Providing Constructive Criticism*) also increased significantly (t = 2.190, p = 0.044) after the workshop. (Table 2) In summary, five of the 13 total post-course survey items were shown to have increased at statistically significant levels from pre-course levels.

### Post-Course Evaluations

Eleven additional items in the post-course survey were included to ask respondents to evaluate different aspects of the on-line feedback and in-class workshop components of the curriculum. In summary, at least 64% of respondents reported either *Agree* or *Strongly Agree* when positively evaluating parts of the feedback course on a possible five point Likert-type scale from “1” “*Strongly Disagree”* to”5” “Strongly *Agree”* scale. Approximately five (26%) participating faculty reported some degree of difficulty either accessing the online course and/or navigating online course content.

## DISCUSSION

In summary, five of the 13 total post-course FFS^7^ survey items increased at statistically significant levels from pre-course levels. Similar to several earlier GME studies,^14,15,17^ the majority of participating faculty comments also positively evaluated the flipped curriculum online content and workshop activities.

Readers should consider these initial pilot results within the context of several clear project limitations. The results came from a smaller convenience sample of 17 GME faculty who already appeared from pre-course survey responses to be fairly satisfied and/or comfortable with their resident feedback behaviors. The project was certainly likely underpowered to detect some meaningful pre-post-course sample subgroup differences (e.g. male versus female residents) that might be detectable in a larger diverse faculty sample. The authors acknowledge that measured increases in participants’ feedback satisfaction and confidence ratings may have been somewhat skewed by some degree of *Hawthorne/observer effect* since respondents knew their study responses would be reviewed by the authors.

## CONCLUSION

These promising project findings suggest that a flipped GME faculty-training model may be useful to improve faculty feedback satisfaction and comfort when supervising residents and/or medical students. The full impact of these types of curricular models for GME faculty requires studies more rigorously examined in settings with larger samples to tease out what specific types of curricular activities might be most effective for the diverse faculty learners.

In hindsight, the authors could have more aggressively tried to address and discuss perceived barriers to providing resident feedback for faculty during the online pre-course training content. The authors could have also increased in-class feedback training activities to provide faculty with more opportunities to apply this content in a variety of hypothetical scenarios.

These measured improvements in faculty learners’ perceived feedback satisfaction and comfort provide evidence suggesting that the more purposeful targeting and delivery of such educational curricula may contribute to community-based GME faculty development programs. Ideally, the curricular refinements developed from future studies will lead to improvements in the feedback patterns of GME faculty supervising residents and medical students across the nation.

### Conflict of Interest

The authors declare no conflict of interest.
